# Inhibition of the C-X-C Motif Chemokine 12 (CXCL12) and Its Receptor CXCR4 Reduces Utero-Placental Expression of the VEGF System and Increases Utero-Placental Autophagy

**DOI:** 10.3389/fvets.2021.650687

**Published:** 2021-08-16

**Authors:** Ryan L. Ashley, Cheyenne L. Runyan, Marlie M. Maestas, Elisa Trigo, Gail Silver

**Affiliations:** Department of Animal and Range Sciences, New Mexico State University, Las Cruces, NM, United States

**Keywords:** CXCL12, CXCR4, CXCR7, angiogenesis, placenta, MAPK, fetal/maternal crosstalk, autophagy

## Abstract

The placenta, a unique organ that only develops during pregnancy, is essential for nutrient, oxygen, and waste exchange between offspring and mother. Yet, despite its importance, the placenta remains one of the least understood organs and knowledge of early placental formation is particularly limited. Abnormalities in placental development result in placental dysfunction or insufficiency whereby normal placental physiology is impaired. Placental dysfunction is a frequent source of pregnancy loss in livestock, inflicting serious economic impact to producers. Though the underlying causes of placental dysfunction are not well-characterized, initiation of disease is thought to occur during establishment of functional fetal and placental circulation. A comprehensive understanding of the mechanisms controlling placental growth and vascularization is necessary to improve reproductive success in livestock. We propose chemokine C-X-C motif ligand 12 (CXCL12) signaling through its receptor CXCR4 functions as a chief coordinator of vascularization through direct actions on fetal trophoblast and maternal endometrial and immune cells. To investigate CXCL12–CXCR4 signaling on uteroplacental vascular remodeling at the fetal–maternal interface, we utilized a CXCR4 antagonist (AMD3100). On day 12 post-breeding in sheep, osmotic pumps were surgically installed and delivered either AMD3100 or saline into the uterine lumen ipsilateral to the corpus luteum for 14 days. On day 35 of ovine pregnancy, fetal/placental and endometrial tissues were collected, snap-frozen in liquid nitrogen, and uterine horn cross sections were preserved for immunofluorescent analysis. Suppressing CXCL12–CXCR4 at the fetal–maternal interface during initial placental vascularization resulted in diminished abundance of select angiogenic factors in fetal and maternal placenta on day 35. Compared to control, less vascular endothelial growth factor (VEGF) and VEFG receptor 2 (KDR) were observed in endometrium when CXCL12–CXCR4 was diminished. Less VEGF was also evident in fetal placenta (cotyledons) in ewes receiving AMD3100 infusion compared to control. Suppressing CXCL12–CXCR4 at the fetal–maternal interface also resulted in greater autophagy induction in fetal and maternal placenta compared to control, suggestive of CXCL12–CXCR4 impacting cell survival. CXCL12–CXCR4 signaling may govern placental homeostasis by serving as a critical upstream mediator of vascularization and cell viability, thereby ensuring appropriate placental development.

## Introduction

Reproductive losses due to embryonic and fetal deaths result in crucial economic constraints to livestock and dairy production systems and also manifest in loss of genetically important animals. Many pregnancy losses occur due to complications that arise when the embryo attaches to maternal endometrium (implantation) and subsequent formation of the placenta (placentation). The placenta, a unique organ that only develops during pregnancy, is essential for nutrient, oxygen, and waste exchange between offspring and mother. Abnormalities in placental development result in placental dysfunction or insufficiency whereby normal placental physiology is impaired. Placental insufficiency is a frequent source of pregnancy loss in livestock, inflicting serious economic impact to producers (1, 2), but the underlying causes of impaired placental function are not well-characterized. Placental dysfunction is at the root of numerous pregnancy complications such as pre-eclampsia and intrauterine growth restriction (IUGR). These pregnancy complications are the leading cause of maternal, fetal, and neonatal morbidity and mortality worldwide in humans. Impaired placental function pre-disposes offspring to cardiovascular disease, type 2 diabetes, insulin resistance, obesity, hypertension, and stroke during adulthood (3–8). Though less studied in livestock, these same health problems occur in farm animals, negatively affecting livestock production (9). A comprehensive understanding of the mechanisms controlling placental growth and vascularization are necessary to improve reproductive success in livestock and dairy systems.

Despite its importance, the placenta remains one of the least understood organs and knowledge of early placental formation is particularly limited. Placental development requires intricately coordinated communication between fetal trophoblast and maternal endometrial and immune cells at the fetal–maternal interface during a defined window early in gestation. Numerous factors including chemokines, cytokines, and growth factors contribute to the dialog at the fetal–maternal interface to direct placental development (10, 11). Among these, the unique chemokine network, more known for immune system-specific functions, has emerged as a critical player in regulating implantation and placentation. Chemokines constitute a family of small (8–14 kDa) structurally similar peptides that elicit actions by binding and activating various G protein-coupled receptors. Chemokine ligand 12 (CXCL12) also known as stromal cell-derived factor (SDF-1) is involved in several processes that are also central to placentation including stimulation of cell proliferation and migration, vascularization, immune cell recruitment, and cytokine production through direct actions on fetal trophoblast and maternal endometrial and immune cells (12). As CXCL12 acts directly upon fetal trophoblast and maternal endometrial and immune cells, all of which coordinate placentation, it has great potential to generate a supportive placental microenvironment, thereby directing placental formation. However, the precise roles that CXCL12 plays in livestock placental development are limited.

Using an *in vivo* sheep model in combination with *in vitro* methods, we have gained insight into CXCL12-induced actions during conceptus attachment and initial placentation. Suppressing CXCL12/CXCR4 signaling during the small window of conceptus implantation diminishes placental vascularization, induces autophagy, and dampens the inflammatory placental environment (13–16). Moreover, we recently published evidence demonstrating that suppressing CXCL12-induced actions at the fetal–maternal interface reduces trophoblast invasion into maternal endometrium and delays uterine remodeling (13). Notably, several observed outcomes in our studies mirror those of placental dysfunction, suggesting that an imbalance in CXCL12-mediated signaling may be causative. Building on these studies, we hypothesized that the reduced vascularization, diminished trophoblast invasion, and altered uterine receptivity observed upon suppressing CXCL12–CXCR4 during attachment would manifest in compromised placentation later in gestation. To test, we investigated impact to placental vascularization, cell survival, and relevant signaling pathways in the placenta on d35 of gestation after suppressing CXCL12–CXCR4 signaling at the fetal–maternal during implantation. Day 35 of pregnancy was selected to represent a time when the conceptus should be firmly attached, and uteroplacental vascular remodeling is rampant (17–19). We suggest that CXCL12 signaling through its receptor CXCR4 functions as a chief coordinator of placentation through direct actions on fetal trophoblast and maternal endometrial cells. The goal is to increase understanding of CXCL12-induced actions on controlling placental growth and vascularization to improve reproductive success and fetal health in livestock and dairy production systems.

## Materials and Methods

### Animals

All procedures involving animals were conducted with approval by the New Mexico State University Institutional Animal Care and Use Committee. Fifteen Western white face ewes (primarily Rambouillet, Targhee, and Columbia), similar in age (3–5 years) and weight, received intravaginal controlled internal drug release (CIDR) inserts for 5 days to synchronize estrus, and upon removal, two injections of dinoprost tromethamine (5 mg intramuscular; Lutalyse; Pfizer, New York, NY) were administered 4 h apart (14). Ewes were mated by a fertile ram and randomly placed into experimental groups of either control (PBS; *n* = 7) or treatment (CXCR4 inhibitor; AMD3100; *n* = 8). On day 12 of gestation, ewes were anesthetized (5 mg xylazine and 100 mg ketamine, 1 ml intravenous) and maintained on isoflurane. Mini-osmotic pumps developed for 14-day delivery (2 ml reservoir volume and pumping rate of 5 μl/h; Alzet 2ML1, Cupertino, CA, USA) were pre-loaded with AMD3100 (2,060 ng; Selleckchem, Houston, TX, USA) or PBS. The AMD3100 dose is based on the pharmacokinetic profiling of AMD3100 and median values reported in circulation (20). The catheter attached to the pump was inserted into the lumen of the uterus ipsilateral to the corpus luteum, thus dispensing treatments into the uterine lumen. The pump and catheter were anchored to the uterus with cyanoacrylate glue (super glue, Ontario, CA) and secured with suture (MWI Vet Supply, Boise, ID. USA).

### Tissue Collections

On day 35 of gestation, ewes were anesthetized with sodium pentobarbital (20 mg/kg, intravenous) and reproductive tract was removed using a mid-ventral laparotomy. Endometrial (caruncle and intercaruncle) and cotyledon tissues were collected, snap-frozen in liquid nitrogen, and stored at −80°C for subsequent protein isolation. Using a sterile razor blade, frontal sections (0.5 cm thick) of the ipsilateral uterine horn were also collected and immersed in 4% paraformaldehyde for 24 h. Uterine horn cross sections were embedded in paraffin, sectioned at 5 μm, and mounted onto glass slides using standard procedures (AML Laboratories). Ewes that did not have fetal membrane or embryos present at time of tissue collection were designated as non-pregnant. Ewes were euthanized by exsanguination while under anesthesia.

### Protein Isolation and Immunoblot

Protein was isolated from endometrial and cotyledon tissues of all pregnant ewes by homogenizing 100 mg of tissue in 1 ml of RIPA buffer supplemented with phosphatase and protease inhibitor tablets. Protein samples were placed on ice for 15 min and centrifuged at 12,000 × g for 10 min at 4°C before supernatant was removed and lysates were stored at −80°C until further analysis. Protein lysate concentrations were quantified using BCA protein assay (Thermo Fisher Scientific). Equal amounts of protein lysate were separated using SDS-PAGE followed by transfer to methanol-activated polyvinyl difluoride (PVDF) membranes for immunoblotting. All washes and dilutions were made in TBS supplemented with 0.10% TWEEN 20, unless otherwise noted. Membranes were blocked in either 5% non-fat milk or 5% BSA for 1 h at room temperature, followed by incubation with primary antibody diluted in either 5% non-fat milk or 5% BSA overnight at 4°C. Antibody specifications are as follows: vascular endothelial growth factor (VEGF; 1:500; sc-152; Santa Cruz Biotechnology), C-X-C chemokine receptor type 7 (CXCR7; 1:500; NBP2024779, Novus Biologicals), kinase insert domain receptor/VEGF Receptor 2 (KDR; 1:1,000; NB100-627, Novus Biologicals), microtubule-associated proteins 1A/1B light chain 3B (LC3B-II; 1:1000; PA1-46286; Thermo Fisher Scientific), phosphorylated extracellular signal-regulated kinase 1/2 (pERK; 1:1,000, sc93, Santa Cruz Biotechnology), extracellular signal-regulated kinase 1/2 (ERK; 1:1,000; sc7383; Santa Cruz Biotechnology), phosphorylated c-Jun N-terminal Kinase (pJNK; 1:1,000; cst 9252; Cell Signaling Technology), c-Jun N-terminal Kinase (JNK; 1:1,000; cst 9251; Cell Signaling Technology), phosphorylated p38 MAP kinase (p-p38; 1:1,000; cst 4511; Cell Signaling Technology), and p38 MAP kinase (p38; 1:1,000; cst 9212; Cell Signaling Technology). After three 10-min washes, membranes were incubated with appropriate horseradish peroxidase (HRP)-conjugated secondary antibody at room temperature for 1 h, followed by two final 10-min washes and subsequent incubation with chemiluminescent HRP substrate (34076; Thermo Fisher Scientific). Proteins of interest were visualized using the ChemiDoc XRS System and Image Lab Software (version 3, Bio-Rad Laboratories), and optical densitometry values of each band were recorded. Equal loading of all protein samples was validated using an antibody specific to glyceraldehyde phosphate dehydrogenase (GAPDH).

### Immunofluorescence

Paraffin-embedded tissues were sectioned at 5 μm, mounted onto glass slides, and de-paraffinized using a histologic clearing agent (Histo-clear, National Diagnostics, Atlanta, GA) followed by a series of rehydration ethanol washes (100, 95, 70, and 50% ethanol, respectively). Antigen retrieval was performed by boiling samples in 10 mM sodium citrate buffer, pH 6, in a microwave for 10 min, and each slide was rinsed twice in TBS with 0.025% Triton X-100. To prevent non-specific binding of antibodies, each slide was treated for 1 h with blocking buffer (TBS + 1% BSA + 10% normal goat serum). Tissue sections were incubated with a specific primary antibody specific to von Willebrand factor (vWF; cat no. ab6994; Abcam). Slides were rinsed 2 × 5 min in TBS-T and incubated for 1 h at room temperature with Alexa 568-labeled goat anti-rabbit secondary antibody. Each slide was mounted with Fluoromount (Sigma-Aldrich) with 4,6-diamidion-2-phenylindole (Life Technologies, Grand Island, NY) to counterstain nuclei. Control sections were incubated with dilution buffer (TBS + 1% BSA).

### Microscopy Image Analysis

For each tissue section, photomicrographs were taken at 10× at the same exposure time with Zeiss Axio Scope and AxioCam MRm camera for immunofluorescence (Carl Zeiss Microscopy, LLC, Thornwood, NY). Using this method, we obtained images of placental and gravid uterus vasculature that can be quantified. Blood vessel abundance was analyzed as previously described (21). Each level was scanned for blood vessels and imaged. Within each level, endothelial cell marker, vWF expression, blood vessel number, and blood vessel circumference were analyzed.

### Statistical Analysis

Significant differences in immunoblot data were determined by performing an unpaired, two-tailed Student's *t*-test analysis using Prism (version 9, GraphPad Software, Inc.). Chemiluminescent signals for Western blots were quantified using the Image Lab software program; the mean intensity value was obtained for each band of interest and normalized by dividing that of the corresponding band for GAPDH. Immunofluorescent signals from uterine cross sections were quantified using the ImageJ software program; the mean integrated density values were obtained and averaged. Tissue autofluorescence was accounted for by measuring the integrated density of the background, averaging the values from each image, and subtracting from the average integrated density of that animal. Values were quantified using ImageJ, and the average integrated density value, area circumference, and blood vessel number were analyzed using the unpaired Student's *t*-test.

## Results

**Figure 1:**
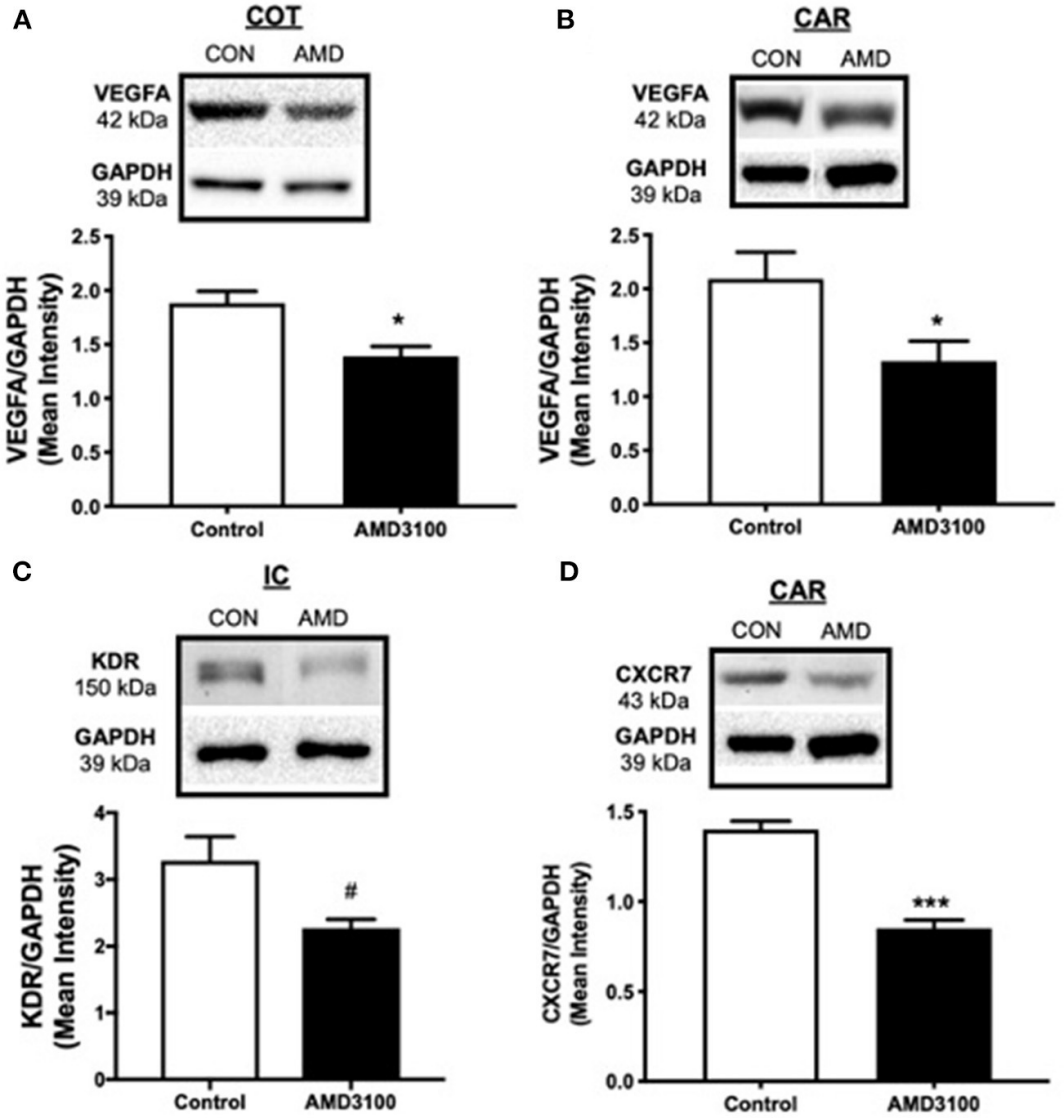
Impact to placental vascularization after suppressing the CXCL12–CXCR4 network. Protein abundance and representative immunoblots of VEGFA in ovine fetal cotyledon (COT) placenta **(A)** and maternal caruncle (CAR) placenta **(B)**, the VEGFA receptor KDR in intercaruncle (IC) tissue **(C)**, and CXCR7 in caruncle placenta **(D)** following intrauterine saline (CON) or AMD3100 (AMD) infusion. Data are presented as the mean ± SEM, and significance is denoted with an asterisk (*) when *p* < 0.05, or (***) when *p* < 0.0001. ^#^*p* = 0.08.

To evaluate placental vascularization on day 35 of gestation, angiogenic and growth factors central to placentation were assayed using Western blot. Fibroblast growth factor 2 (FGF2), VEGFA, VEGFB, angiopoietin 1 (ANG1), KDR, fms-like tyrosine kinase-1 (FLT1/VEGFR1), Hypoxia inducible factor alpha (HIF1A), CXCL12, CXCR4, and CXCR7 were detected in cotyledon and caruncle placental tissue on day 35 of gestation but only significant changes in protein amounts are included. Suppressing CXCL12–CXCR4 signaling at the fetal–maternal interface from days 12 to 26 of gestation resulted in less angiogenic factors in the placenta on day 35, 9 days post-treatment (Figure 1). Using immunoblot analysis, protein abundance for VEGFA in both fetal cotyledon (COT) and maternal caruncle (CAR) placenta was diminished (*p* < 0.05) upon suppressing CXCR4 signaling during conceptus attachment (Figures 1A,B). Receptors for VEGF were also assayed and while levels of FLT1 were similar between control and AMD3100-treated ewes in fetal and maternal placenta (data not shown), quelling CXCL12–CXCR4 actions tended (*p* < 0.1) to reduce KDR amounts in intercaruncle (IC) tissue (Figure 1C). Protein expression for CXCR4 did not differ in placenta on day 35 regardless of treatments (data not shown), but protein abundance for the other CXCL12 receptor, CXCR7, was diminished in caruncle placenta when ewes received CXCR4 inhibitor in utero (Figure 1D).

**Figure 2:**
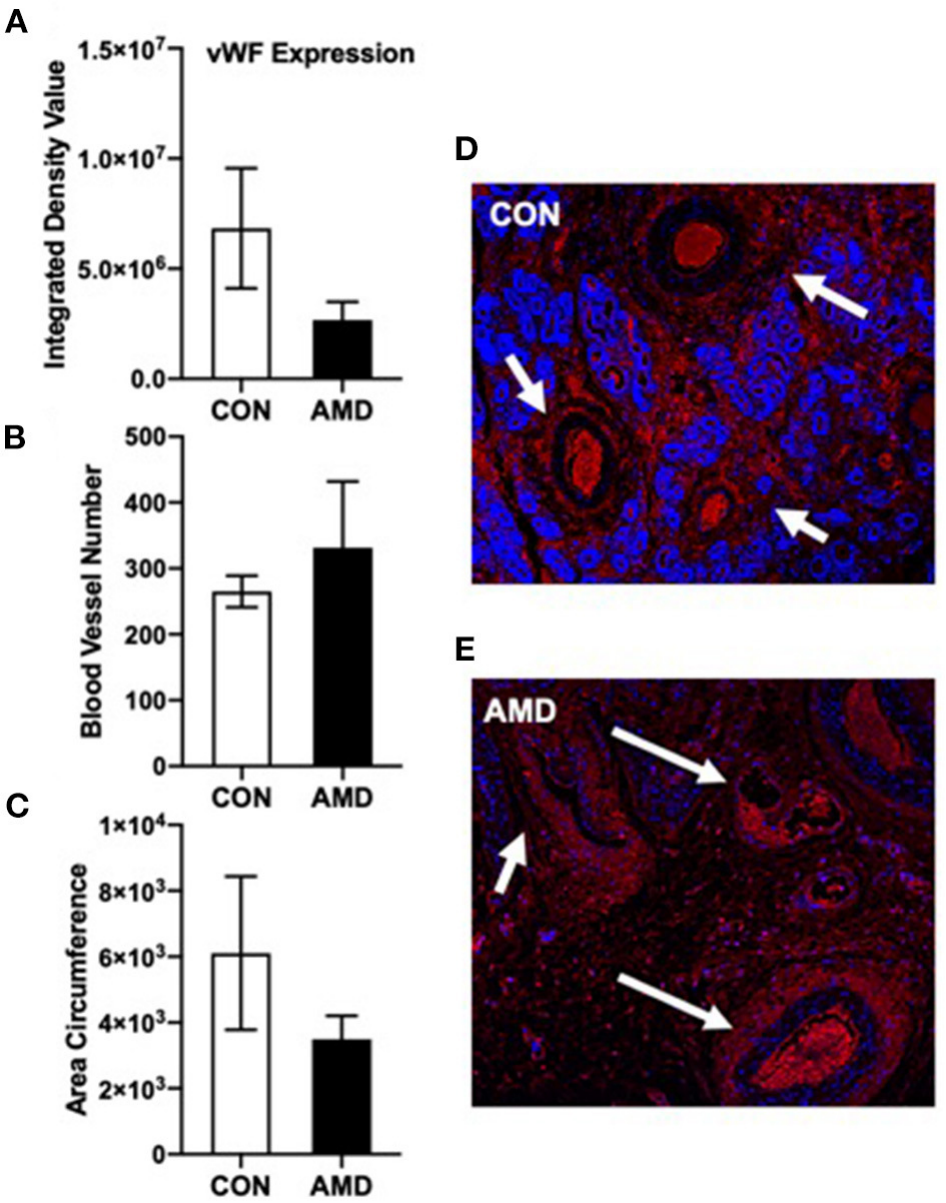
Immunoreactive vWF cells (endothelial cell marker) in ovine uterine cross sections on day 35 of gestation. vWF red fluorescence expression **(A)** was similar (*p* > 0.05) and the number of blood vessels **(B)** and blood vessel circumference **(C)** did not change (*p* > 0.05) between CON and AMD. Representative 10× magnification (100 μm) images of vWF (red) with DAPI (blue) as nuclear stain in ovine uterine horn cross sections following intrauterine treatment with control (CON) phosphate buffer saline **(D)** or CXCR4 inhibitor (AMD) **(E)**. White arrows denote developing blood vessels.

To further determine if vascularization is impacted when CXCL12–CXCR4 signaling is suppressed at the fetal–maternal interface, the expression of vWF was assayed in the placenta using immunofluorescent imaging (Figure 2). Our objectives were to determine immunohistochemical expression of vWF and to evaluate blood vessel number (BVN) and blood vessel circumference (BVC) of the uterine horn on day 35 of gestation. Representative immunofluorescent images of vWF in uterine cross sections from control (CON) and AMD3100-treated (AMD) ewes are shown in Figures 2D,E, respectively. The expression of vWF was similar between control and AMD3100-treated ewes (Figure 2A). Similarly, the number of blood vessels (Figure 2B) and respective circumferences (Figure 2C) did not differ between control and AMD3100-treated ewes on day 35.

**Figure 3:**
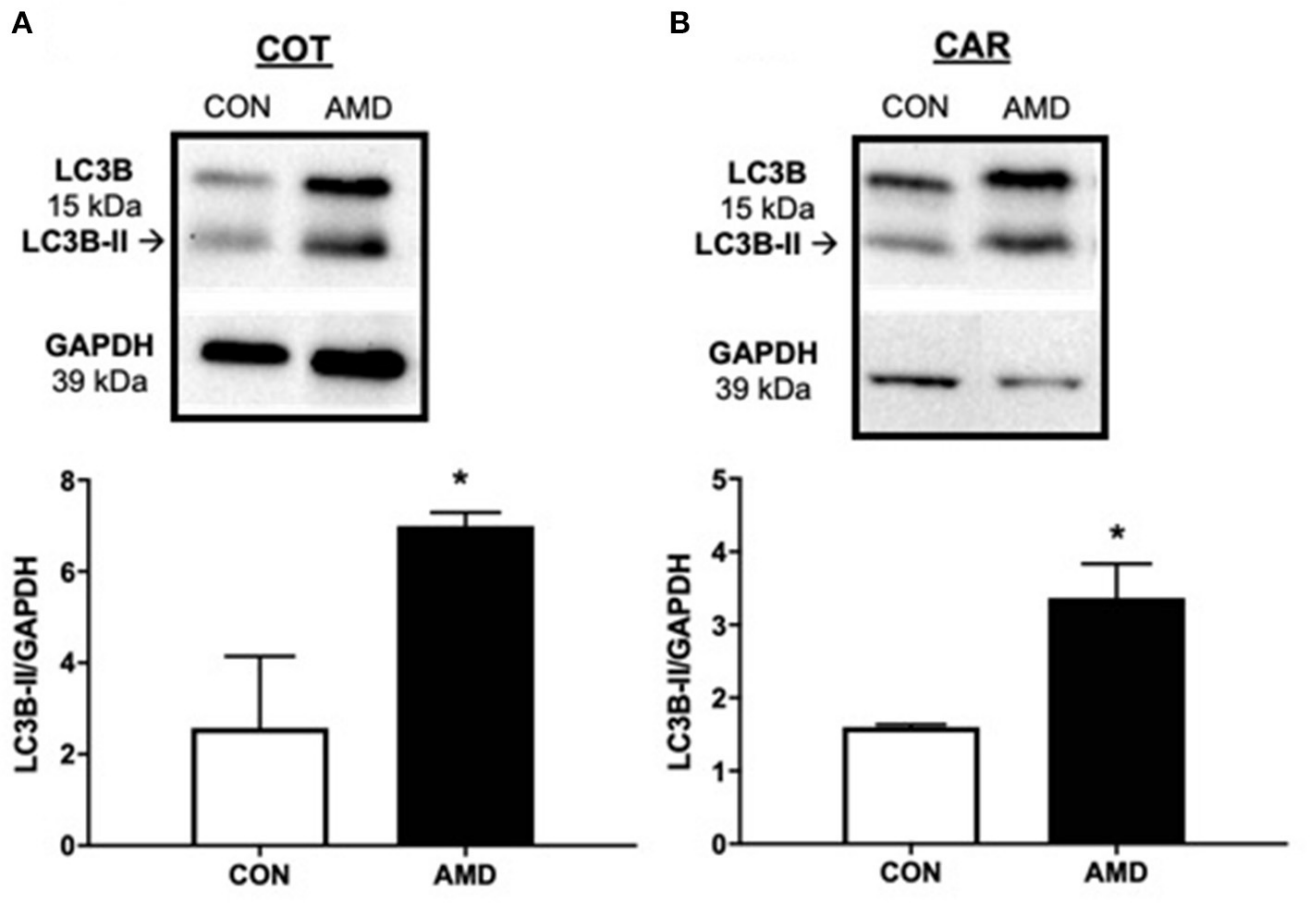
Evidence of placental autophagy induction after suppressing the CXCL12–CXCR4 network. Protein abundance of cellular autophagy marker LC3B-II and representative immunoblots in ovine fetal cotyledon (COT) placenta **(A)** and maternal caruncle (CAR) placenta **(B)** following intrauterine saline (CON) or AMD3100 (AMD) infusion. Data are presented as the mean ± SEM, and significance is denoted with an asterisk when *p* < 0.05 (*).

Induction of autophagy, a cell survival mechanism, was investigated by quantifying protein abundance for LC3B-II, a classic marker of autophagy induction using immunoblot. Compared to control ewes, greater amounts of LC3B-II were evident in both cotyledon (Figure 3A) and caruncle (Figure 3B) placenta from ewes receiving CXCR4 inhibitor during conceptus attachment and beginning stages of placental vascularization.

**Figure 4:**
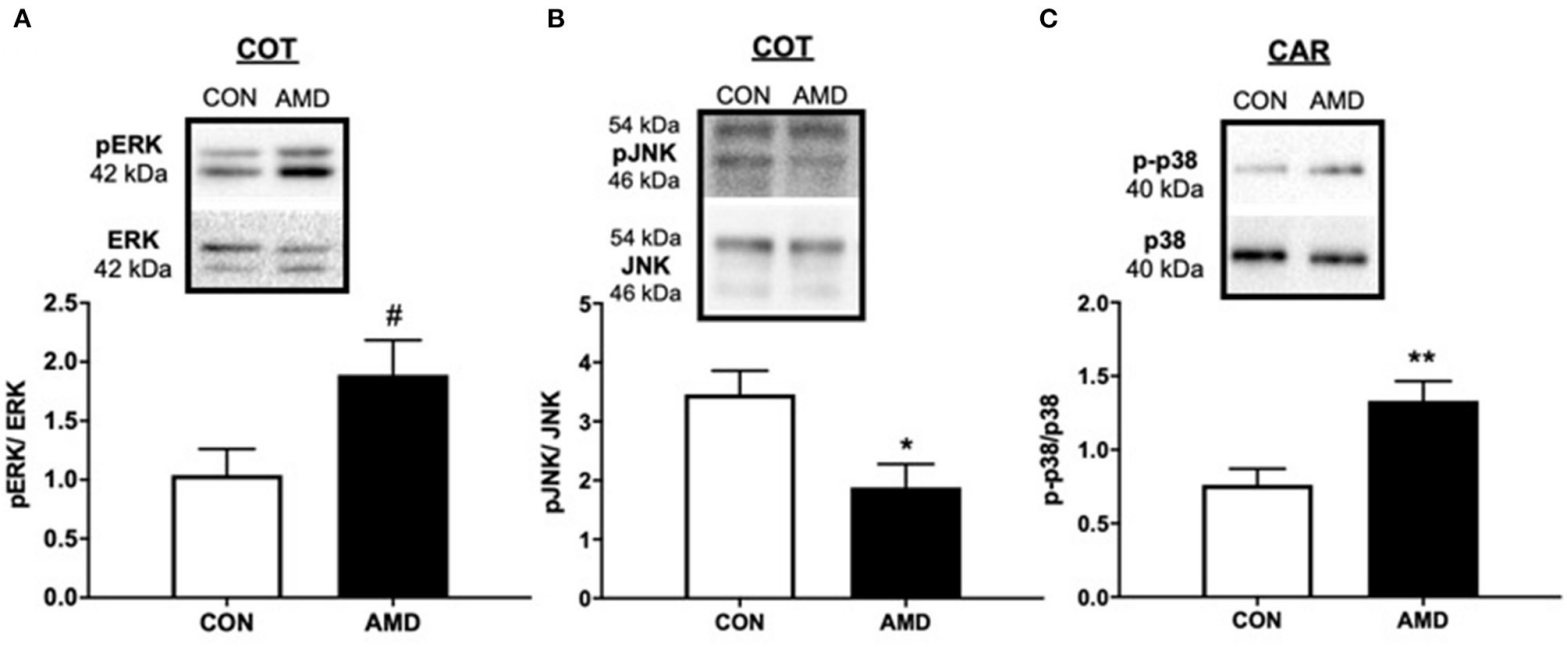
The MAPK-signaling pathways mediated by ERK, JNK, and p38 protein kinases are activated after suppressing the CXCL12–CXCR4 network. Tendency for ERK activation **(A)**, suppression of JNK **(B)** in ovine fetal cotyledon (COT) placenta, and activation of p38 **(C)** in maternal caruncle (CAR) placenta following intrauterine saline (CON) or AMD3100 (AMD) infusion. Phosphoprotein data are shown with corresponding representative immunoblots. Statistical comparisons were made using Student's *t*-test; significance is denoted with an asterisk (*) when *p* < 0.05 or (**) when *p* < 0.01. ^#^*p* = 0.08.

Compared to control ewes receiving saline, all three major MAPKs were altered in placenta 9 days after receiving CXCR4 inhibitor at the fetal–maternal interface (Figure 4). In COT placenta, suppressing CXCL12–CXCR4 resulted in a tendency (*p* = 0.08) for increased ERK activation compared to control as evidenced by the phosphorylated 42- and 44-kDa bands (Figure 4A). The pJNK signaling pathway, however, was diminished (*p* < 0.05) in COT placenta after suppressing CXCR4 at the fetal–maternal interface with less phosphorylated JNK at 46 and 54 kDa (Figure 4B). In maternal placenta (CAR), greater (*p* < 0.05) activation of the p38 pathway was evident after inhibiting CXCL12–CXCR4 during implantation and initial placentation (Figure 4C).

## Discussion

Embryonic and fetal survival is a major factor affecting production and economic efficiency in livestock meat and milk production (22–25) with the majority of losses occurring during implantation and initial placental vascularization (23, 26–30). Placental vascularization is a critical period of early gestation supporting not only embryonic survival but also subsequent fetal growth and development. Diminished placental vascularization can result in placental dysfunction, a fundamental cause of fetal growth abnormalities, pre-eclampsia, and early pregnancy loss (1, 2). The importance of placental circulation to successful pregnancy is recognized and exemplified by the close relationships among fetal weight, placental size, and uterine and umbilical blood flows during normal pregnancies (31–33). Establishment of functional fetal and placental circulations is one of the earliest events during embryonic development (34). The large increase in transplacental exchange, supporting the exponential increase in fetal growth during the last half of gestation, depends primarily on the dramatic growth of placental vascular beds and large increases in uterine and umbilical blood flows early in pregnancy (31–33, 35–39).

Despite clear evidence that placental formation and angiogenesis during early pregnancy are critical for normal fetal growth and development, the underlying molecular mechanisms driving placental vascularization are not well-characterized. Factors influencing placental vascular development drastically impact fetal health, and thus neonatal survival and growth (39–41), whereas compromised fetal growth significantly impacts life-long health and productivity (40, 42, 43). The predominant angiogenic factor driving placental growth and vascularization is vascular endothelial growth factor (VEGFA) signaling through its receptors VEGFA receptor-1 (FLT1) and VEGFA receptor-2 (KDR) (32), yet illuminating central upstream master regulators of the VEGFA angiogenic axis is key to increasing our understanding of placental development.

The CXCL12–CXCR4 chemokine axis has a special relationship with VEGFA and stimulates VEGF production and secretion, which, in turn, drives CXCL12 and CXCR4 synthesis, thereby creating a powerful proangiogenic feed-forward loop (44–47). This angiogenic connection between CXCL12–CXCR4 and VEGFA signaling may be one of the primary pathways promoting early placental vascularization and angiogenesis. Synthesis of CXCL12 and CXCR4 increase in fetal membranes and endometrium prior to VEGFA and VEGFA receptors and the amplified CXCL12–CXCR4 signaling at the fetal–maternal interface may be a central regulator activating the VEGFA angiogenic axis (17, 18, 45, 48). As early as day 18 of pregnancy in sheep, the fetal extraembryonic membranes begin to form blood vessels (31) that coincidently correlates with increased CXCL12 and CXCR4 endometrial expression (45). To determine if CXCL12–CXCR4 signaling is critical to placental production of VEGFA and overall placental vascularization, we developed an *in vivo* model to suppress CXCL12–CXCR4 at the fetal–maternal interface with the CXCR4 antagonist, AMD3100. Suppressing CXCL12–CXCR4 signaling negatively impacts placental vascularization in endometrium as early as day 20 of pregnancy (16) with deleterious impact to placental vascularization still evident 3 days later (15). These data imply that the detrimental impact to placental vascularization when CXCL12–CXCR4 is suppressed may persist, resulting in compromised placental development and negative impact to fetal growth and health later in gestation.

To determine if diminished CXCL12–CXCR4 signaling at the fetal–maternal interface during implantation and initial placental vascularization results in long-term detrimental effects to the placenta, we suppressed CXCL12–CXCR4 at the fetal–maternal interface for 14 days starting on day 12 of gestation (days 12–26) and then collected placental tissues on day 35 of gestation, 9 days after treatment delivery to uterus had stopped. Placental growth, vascularization, cell survival, and relevant signaling pathways were evaluated in fetal and maternal placenta on day 35 of gestation. Similar to previous reports, the negative impact to placental vascularization early in gestation when CXCR4 is antagonized remains evident in placenta several days after treatments subsided. VEGFA abundance was diminished in fetal (COT) and maternal (CAR) placenta on day 35, but unlike our previous studies, FLT1 levels did not differ (15, 16). Instead, production of the other VEGFA receptor, KDR, tended to decrease in maternal placenta after suppressing CXCL12–CXCR4. The current study suppressed CXCL12–CXCR4 signaling at the fetal–maternal interface for 14 days whereas previous studies antagonized CXCR4 for 7 days, which may account for the differences in the production of the different VEGF receptors in the studies. Taken together, it appears that CXCL12–CXCR4 signaling regulates many components of the VEGFA pathway at the fetal–maternal interface including VEGFA and both receptors (15, 16) and thus may be a key upstream regulator of the placental VEGFA angiogenic axis in the placenta.

In an attempt to further quantify placental angiogenesis, we evaluated expression of vWF, as previously used in sheep (49). A large glycoprotein, vWF is produced by endothelial cells and megakaryocytes (50). It is often used as a marker of blood vessels due to its selective endothelial expression and has been used to quantify angiogenesis in a variety of tumors (51–53). Our objectives were to determine immunohistochemical expression of vWF, and to evaluate blood vessel number (BVN) and blood vessel circumference (BVC) of placenta on d35 of gestation. A reduction in vWF was expected because of the reduced VEGFA and associated signaling in placenta in concert with the knowledge that vWF synthesis is upregulated by VEGFA (54). Despite reduced levels of VEGFA in fetal and maternal placenta, vWF remained similar between control and AMD3100-treated ewes. Though not significant, observing vWF immunolocalization, control ewes appeared to have more uniform distribution of vWF and greater vessel circumference compared to AMD3100-treated ewes, which appeared disorganized. It is possible that evaluating vWF on day 35 of gestation is too soon to observe significant changes to placental angiogenesis. Similar to placental dysfunction and pre-eclampsia, the negative impact to placental growth and vascularization may manifest much later in gestation (mid-gestation and onward) after insults during early pregnancy.

The impaired placental vascularization observed on day 35 suggests a stressful placental environment. To determine if overall placental homeostasis was affected, the autophagy pathway was investigated. In addition to degrading cellular components under stress to survive, autophagy also participates in tissue remodeling, growth control, and cellular immunity (55–58), all of which are crucial to proper placental development (59, 60). In the current study, suppressing CXCL12–CXCR4 during implantation and initial placental vascularization resulted in greater abundance of the autophagy marker LC3B-II in fetal and maternal placenta on day 35, consistent with our previous studies (13, 16). However, this is our first observation of autophagy still evident 9 days after treatments had stopped. Whether the main purpose of autophagy induction is survival, tissue remodeling, growth control, or all the above remains to be determined.

To gain a better understanding of the molecular signaling at the fetal–maternal interface upon inhibiting CXCR4, we quantified the MAPK-signaling pathways mediated by ERK, JNK, and p38 protein kinases, which are central to placental growth, survival, and vascularization. The downregulation of p-JNK signaling in fetal (COT) placenta in the current study may indicate a suppression of apoptosis. Given the corresponding rise in autophagy induction observed in the same tissue suggests that these cells are attempting to survive and continue placental development. Moreover, p-ERK tended to increase in COT, which may signify activation of pro-survival and growth signaling mechanisms. Likewise, activation of the p-p38 pathway would indicate that the maternal (CAR) placenta is reacting to a stressful situation and trying to survive. While further studies are needed to determine the downstream consequences to placental development, it is interesting that suppressing a single chemokine receptor, CXCR4 for a brief period results in global changes to several MAPK proteins in the placenta numerous days later in gestation.

CXCR7 is another receptor for CXCL12, though it was only recently demonstrated to elicit intracellular signaling upon CXCL12 binding (61, 62). A study of both receptors at the fetal–maternal interface is needed to illuminate CXCL12-induced actions mediating placental vascularization. While CXCR4 abundance did not change on day 35 after ADM3100 treatment (data not shown), CXCR7 abundance decreased in maternal placenta (CAR) when CXCR4 was inhibited. This is in contrast to our previous studies antagonizing CXCR4 at the fetal–maternal interface where CXCR4 expression is typically altered and CXCR7 remains stable after antagonizing CXCR4 signaling (15, 16). A change to receptor levels suggests possible alterations to CXCL12-induced actions during placental development. The manifestation to placental growth and function later in gestation because of these alterations to CXCL12 receptors during early pregnancy remains to be seen but advocates for further research deciphering roles of CXCR4 and CXCR7 during implantation and placentation.

In conclusion, we demonstrated the impact a short interruption in CXCL12–CXCR4 signaling during implantation has on placental development in sheep. The fact that we can manipulate this chemokine axis during a defined window of early pregnancy with great potential to impact placental development and thus offspring health is exciting and widely applicable to livestock and human health. CXCL12 is a highly conserved chemokine across livestock species and stimulates several biological processes essential to placental development through direct actions on trophoblast, endometrial, and immune cells. As CXCL12 acts upon all cell types responsible for placental formation across eutherian mammals, we propose that modulating CXCL12-induced actions is a novel approach to manipulating the fetal–maternal environment during the window when most pregnancy losses occur, and impaired placental development transpires. Increasing our understanding of CXCL12-induced actions controlling placental growth and vascularization will hopefully reveal methods to improve reproductive success and fetal health in livestock and dairy production systems.

## Data Availability Statement

The raw data supporting the conclusions of this article will be made available by the authors, without undue reservation.

## Ethics Statement

All procedures involving animals were conducted with approval by the New Mexico State University Institutional Animal Care and Use Committee.

## Author Contributions

RA oversaw all of the experimental design, and performed surgical procedures, integrated comments from co-authors, and finalized the manuscript. CR, ET, GS, and MM conducted analyses of protein abundance and provided drafts of manuscript. All authors read and approved the final manuscript.

## Conflict of Interest

The authors declare that the research was conducted in the absence of any commercial or financial relationships that could be construed as a potential conflict of interest.

## Publisher's Note

All claims expressed in this article are solely those of the authors and do not necessarily represent those of their affiliated organizations, or those of the publisher, the editors and the reviewers. Any product that may be evaluated in this article, or claim that may be made by its manufacturer, is not guaranteed or endorsed by the publisher.
