# Supplementary material for: Inhibition of the C-X-C Motif Chemokine 12 (CXCL12) and Its Receptor CXCR4 Reduces Utero-Placental Expression of the VEGF System and Increases Utero-Placental Autophagy
Source: Front Vet Sci. 2021 Aug 16;8:650687. doi: 10.3389/fvets.2021.650687 (PMC8415452; doi:10.3389/fvets.2021.650687)
Supplement: Supplementary file 1 [file Presentation_1.ZIP › d35 Blots_Frontiers paper.pptx]

## Slide 1
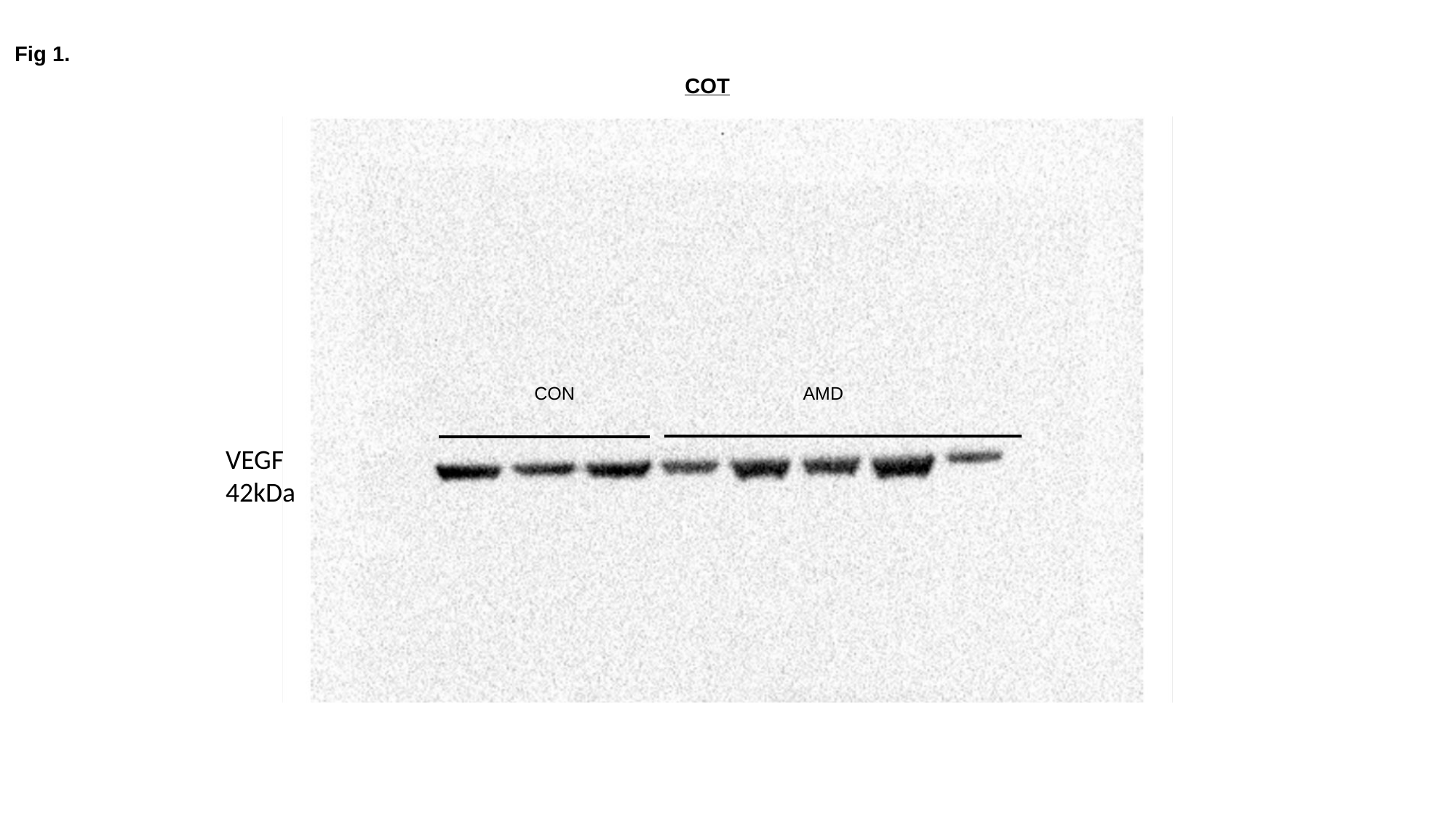

Fig 1.
COT
CON
AMD
VEGF
42kDa

## Slide 2
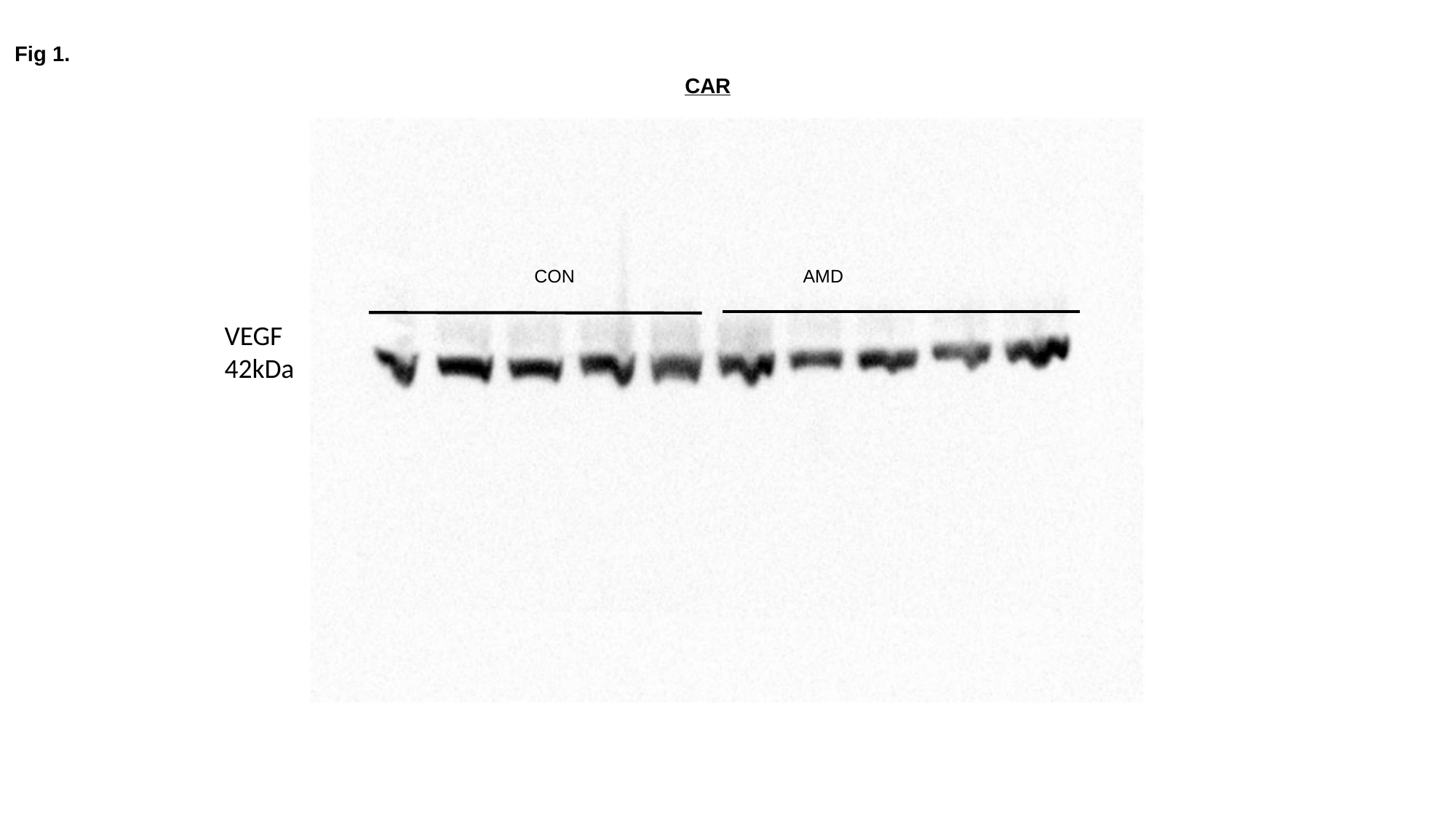

Fig 1.
CAR
CON
AMD
VEGF
42kDa

## Slide 3
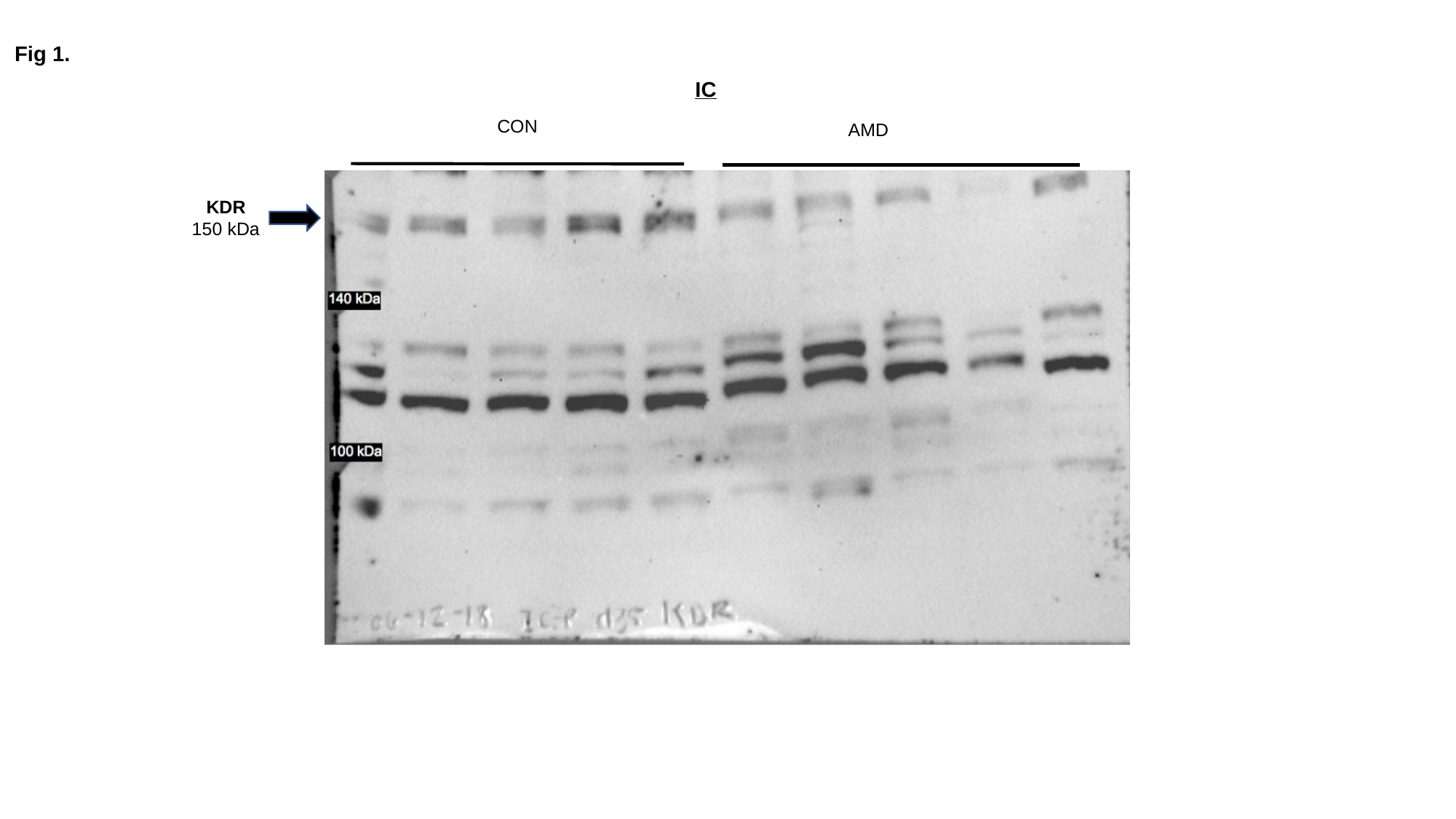

Fig 1.
IC
CON
AMD
KDR
150 kDa

## Slide 4
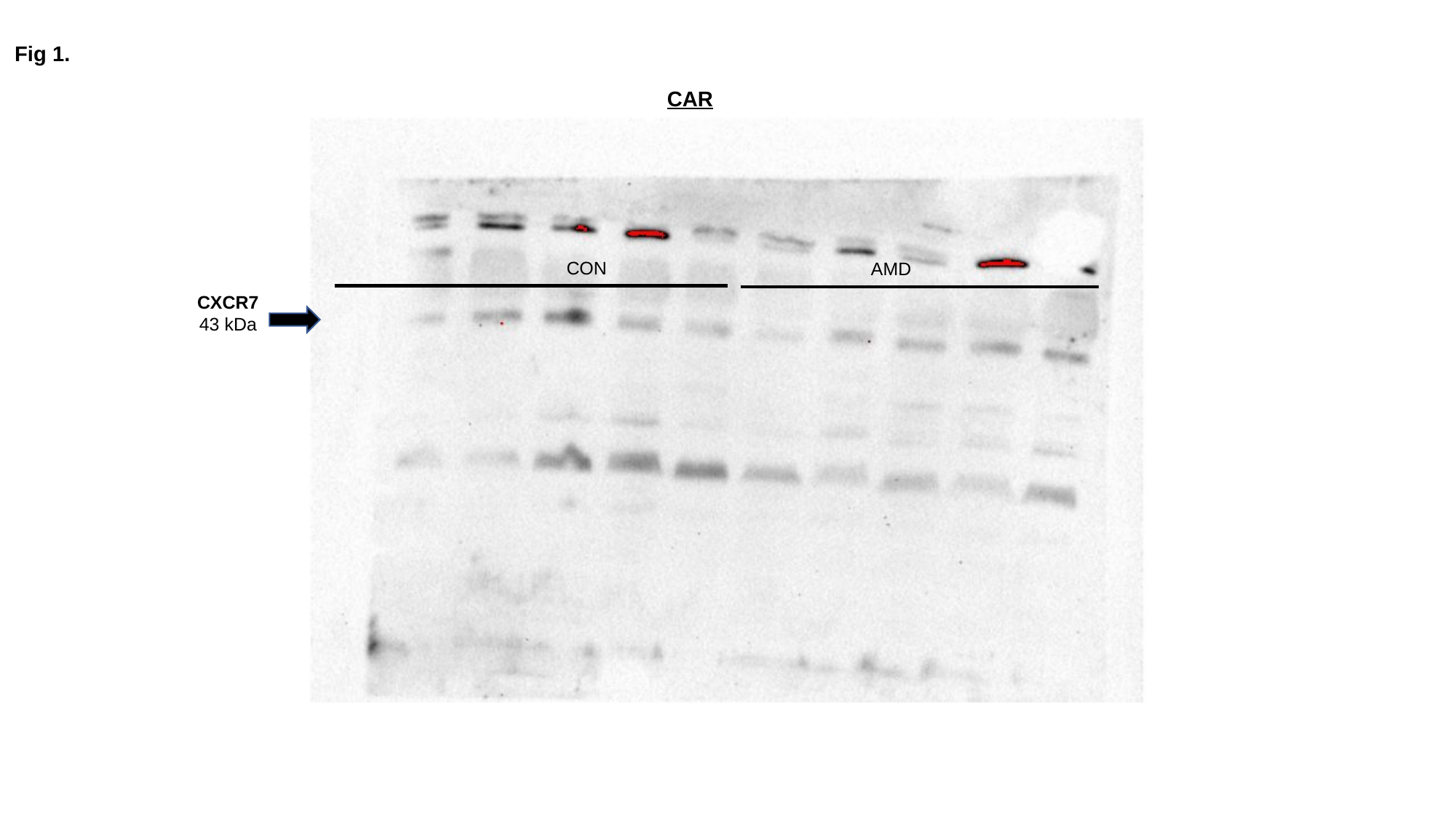

Fig 1.
CAR
CON
AMD
CXCR7
43 kDa

## Slide 5
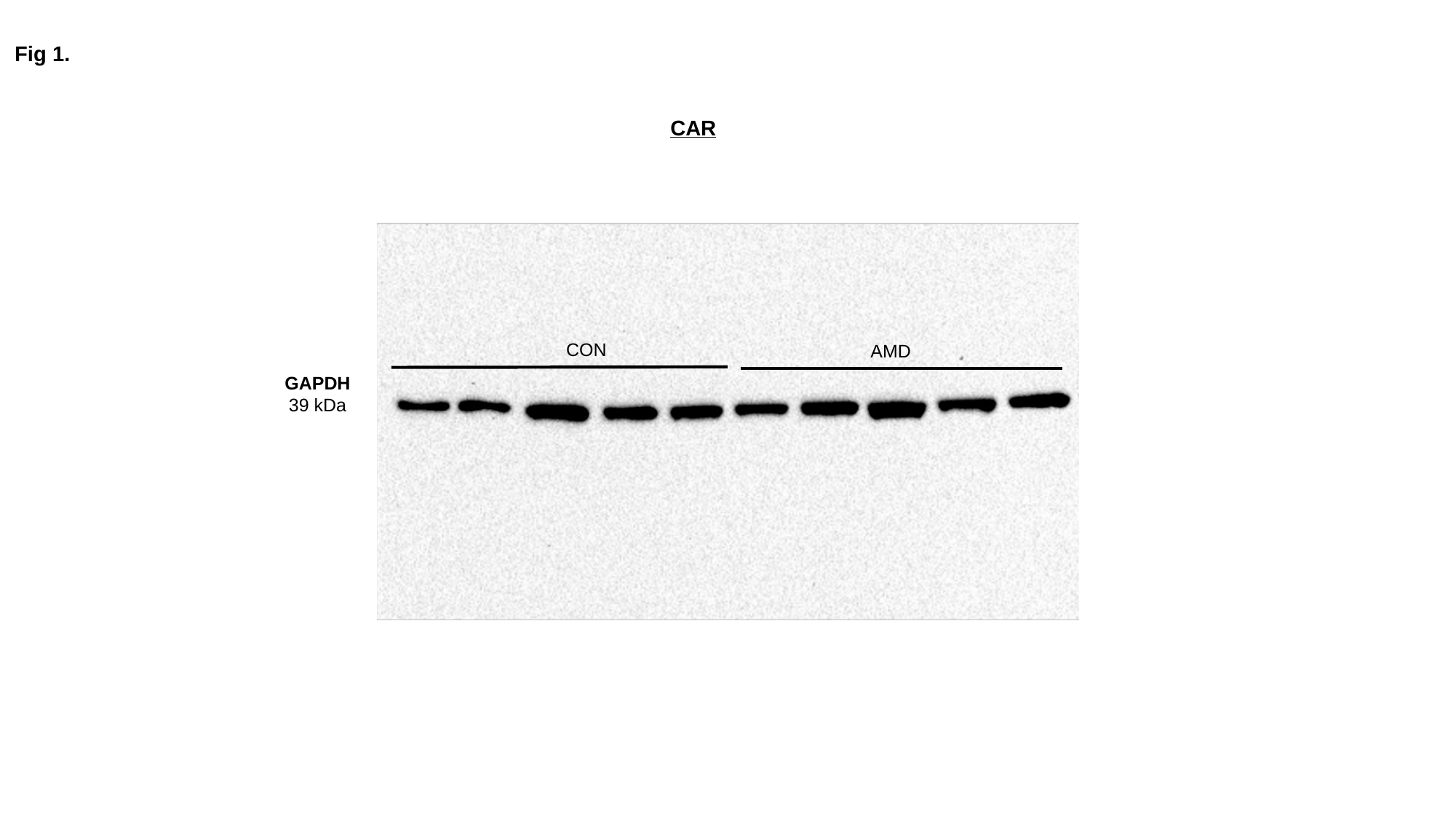

Fig 1.
CAR
CON
AMD
GAPDH
39 kDa

## Slide 6
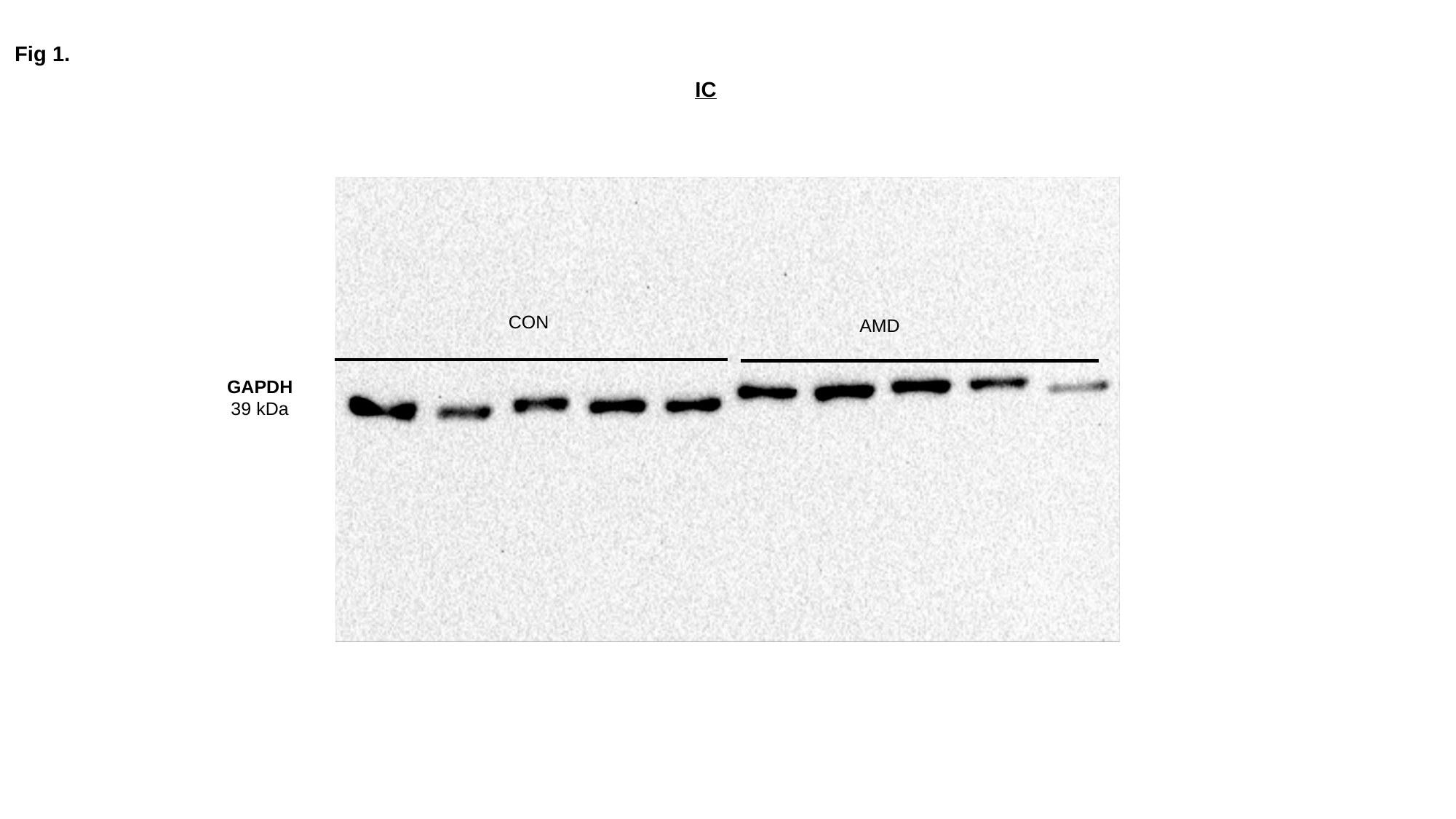

Fig 1.
IC
CON
AMD
GAPDH
39 kDa

## Slide 7
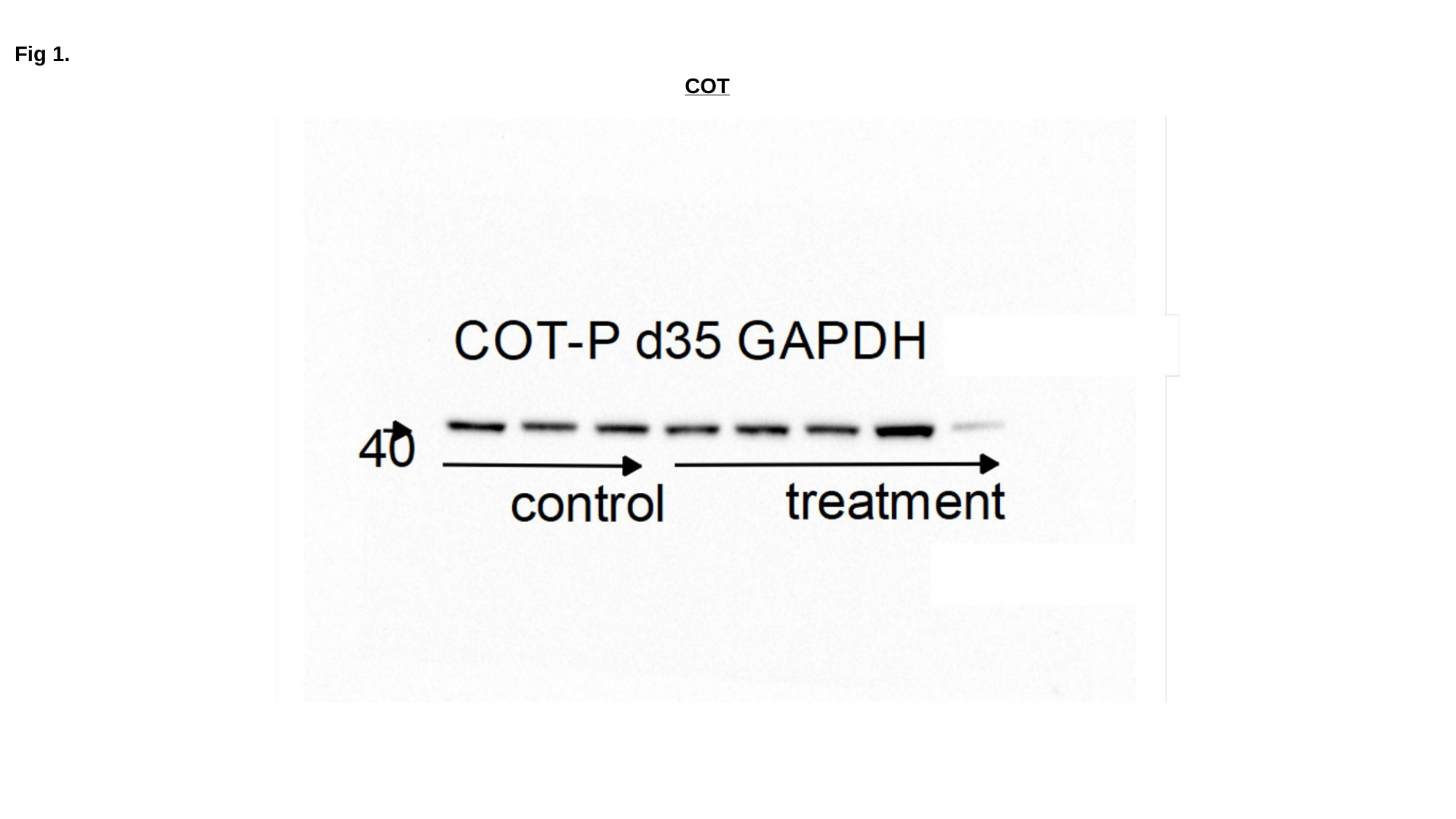

Fig 1.
COT

## Slide 8
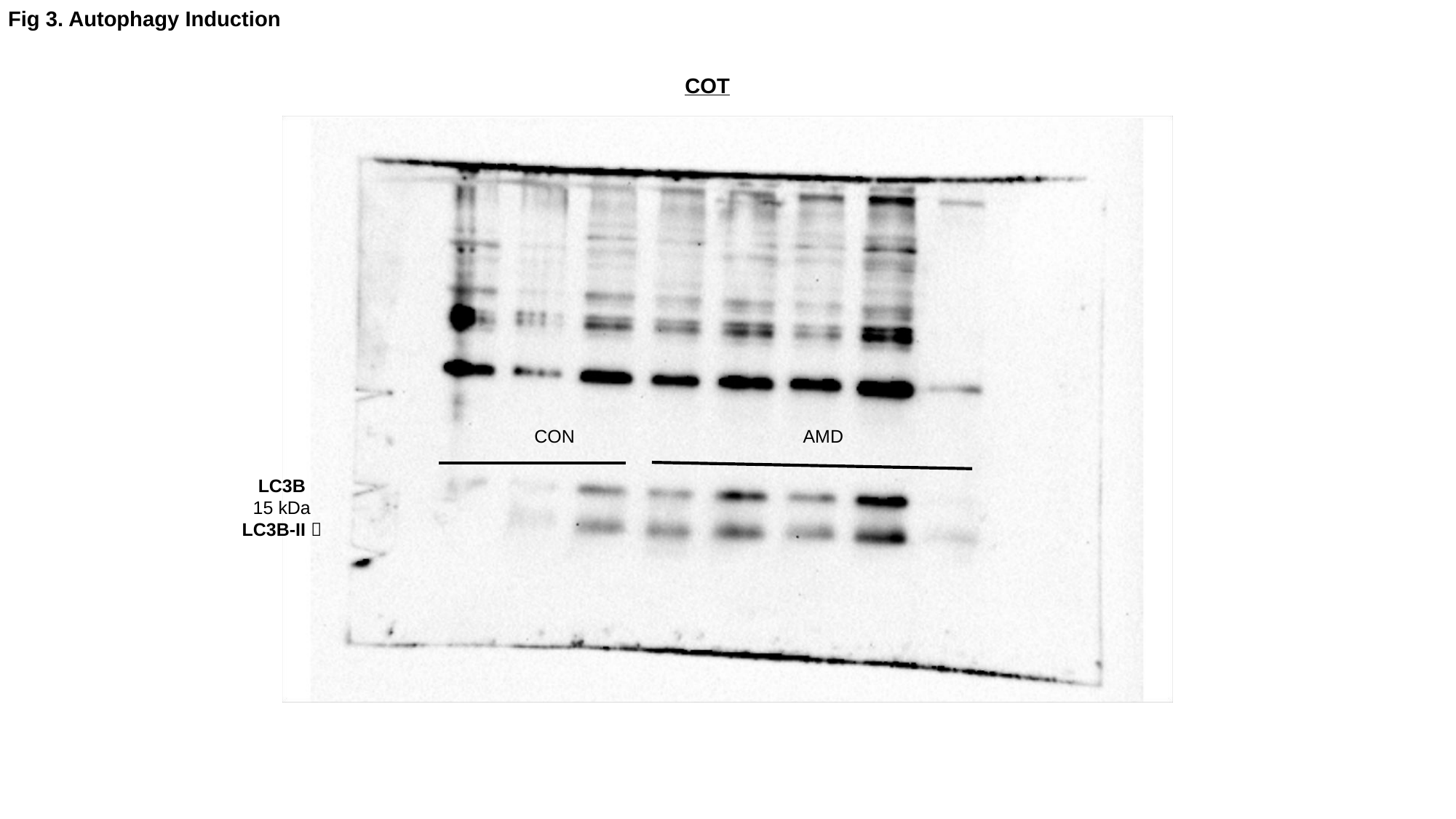

Fig 3. Autophagy Induction
COT
CON
AMD
LC3B
15 kDa
LC3B-II 

## Slide 9
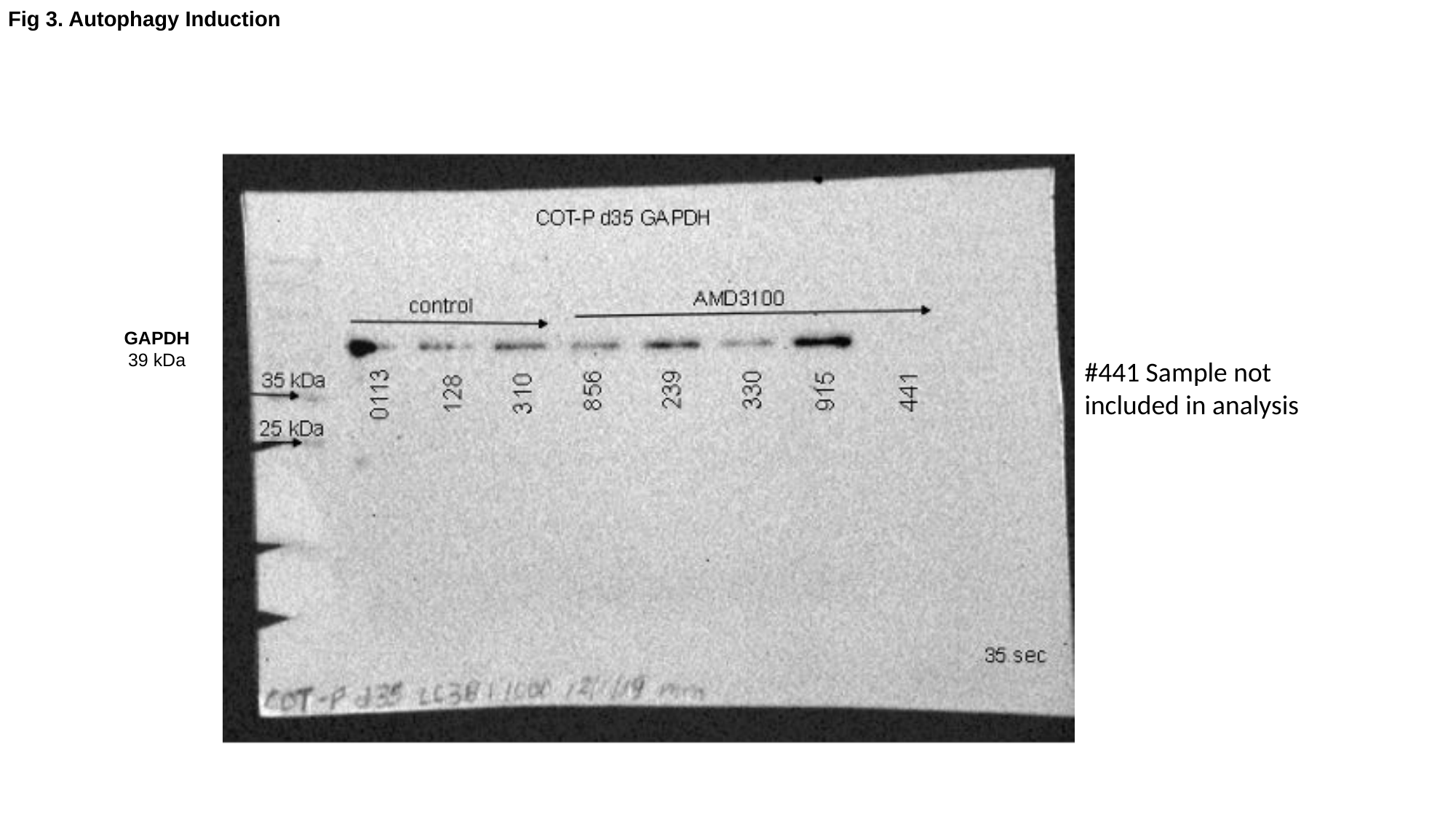

Fig 3. Autophagy Induction
GAPDH
39 kDa
#441 Sample not
included in analysis

## Slide 10
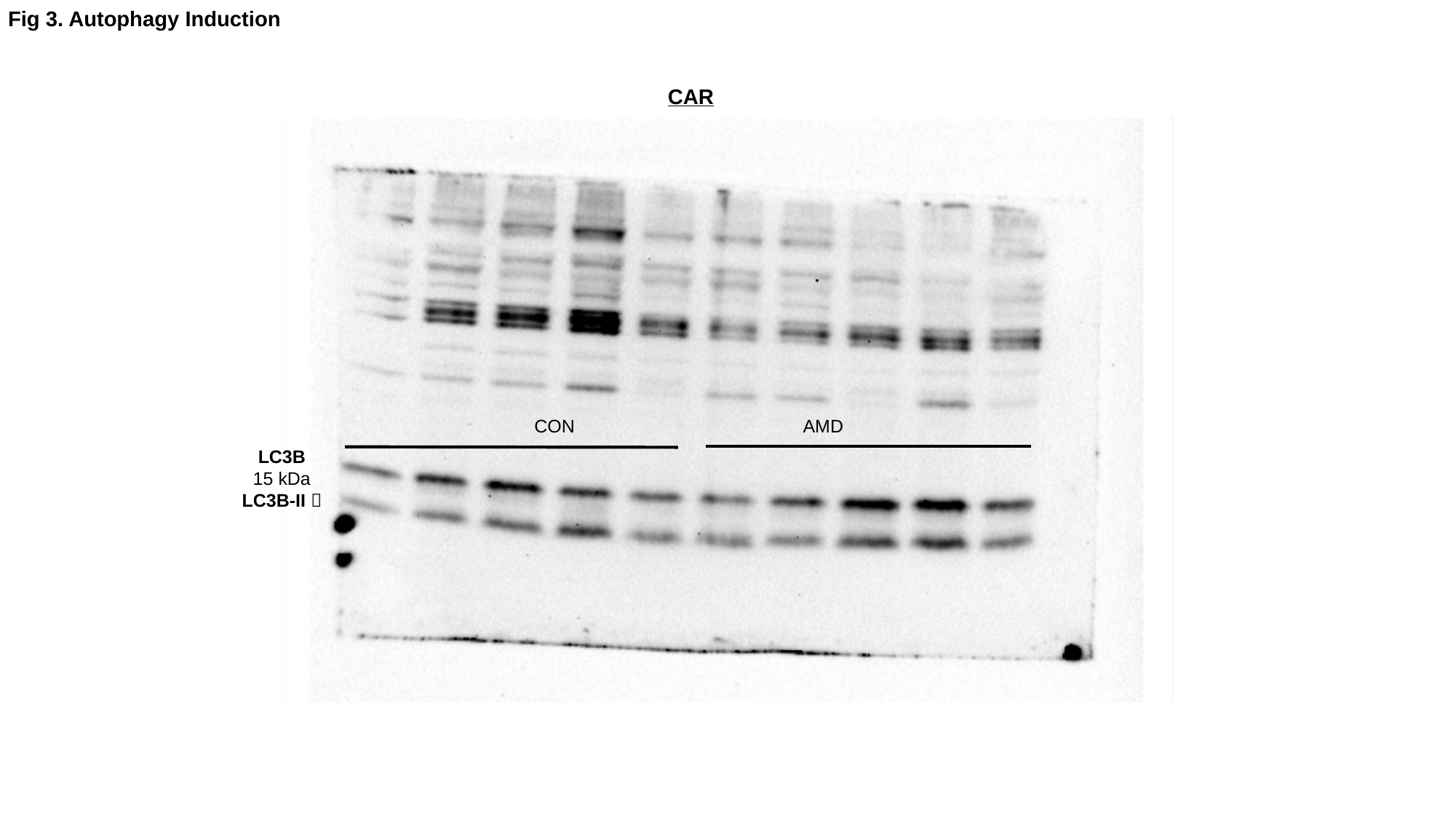

Fig 3. Autophagy Induction
CAR
CON
AMD
LC3B
15 kDa
LC3B-II 

## Slide 11
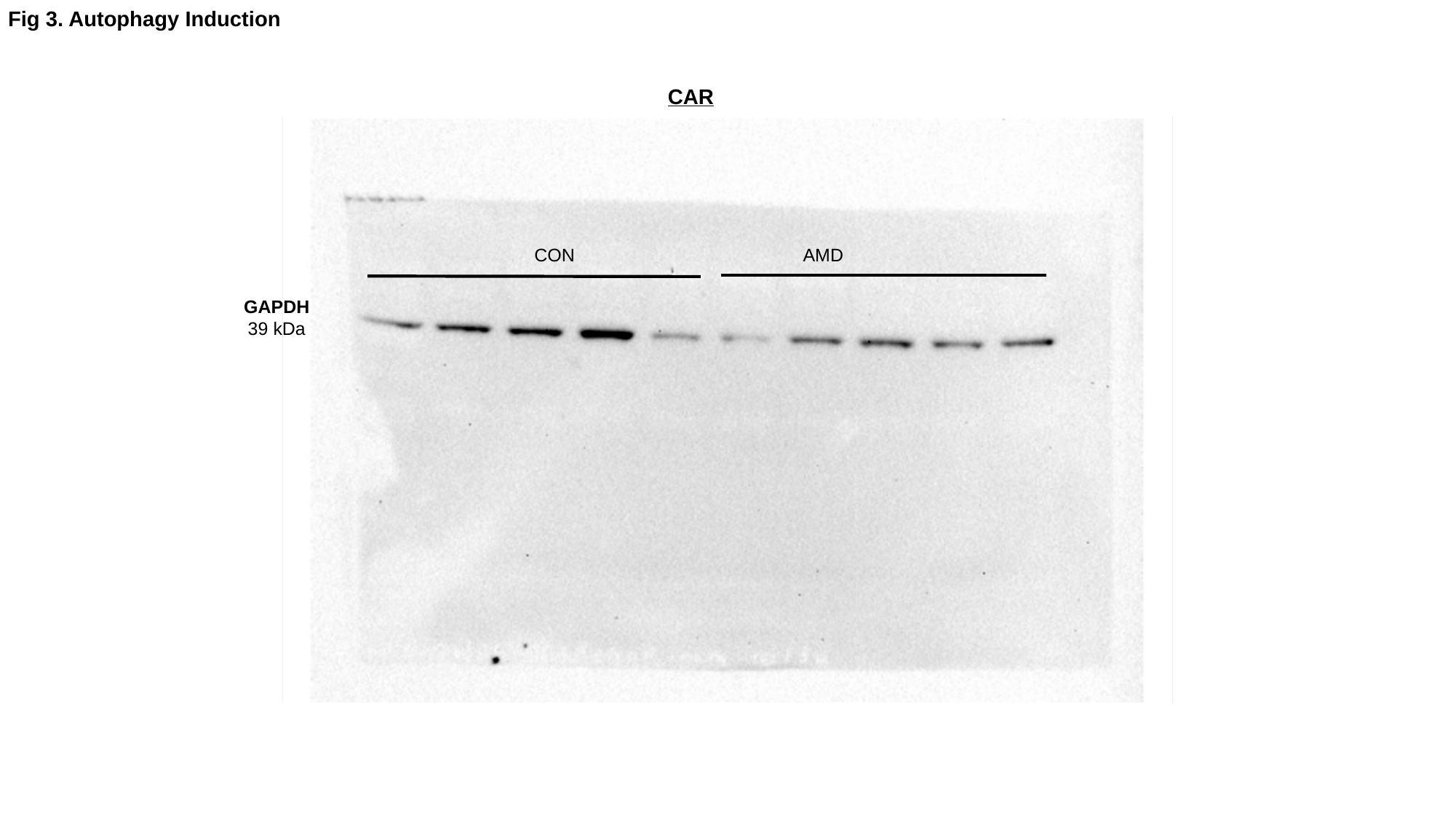

Fig 3. Autophagy Induction
CAR
CON
AMD
GAPDH
39 kDa

## Slide 12
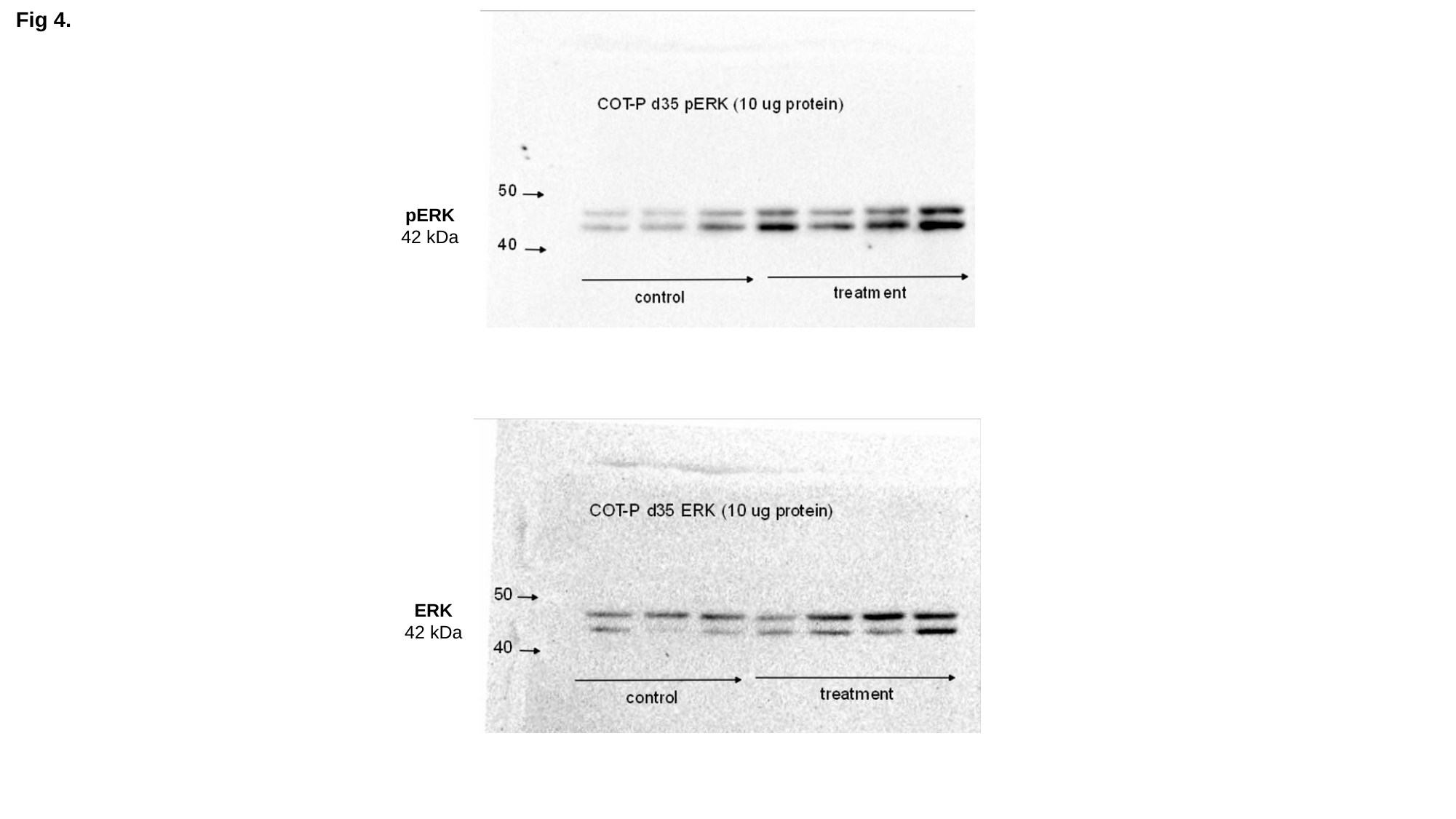

Fig 4.
pERK
42 kDa
ERK
42 kDa

## Slide 13
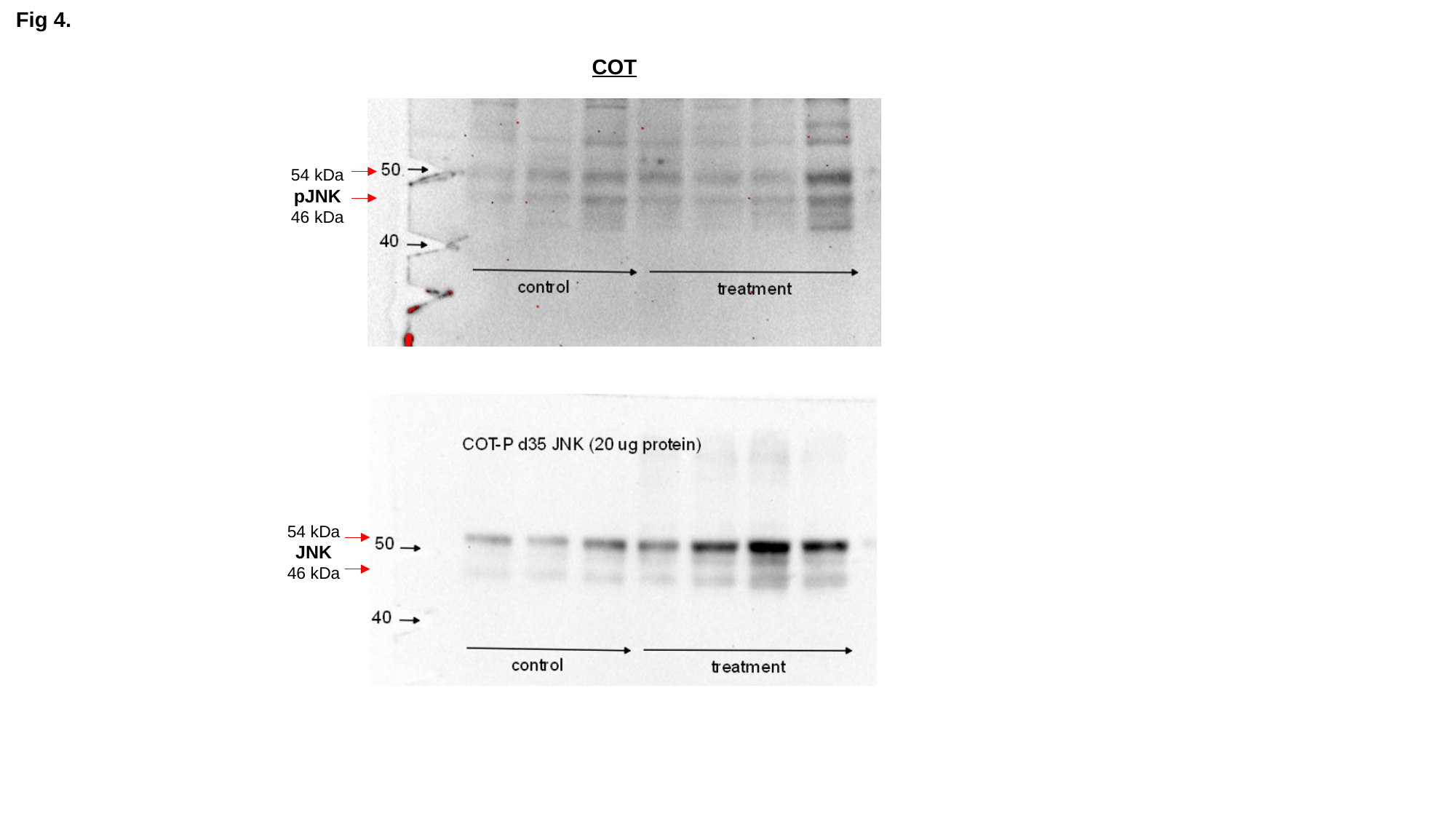

Fig 4.
COT
54 kDa
pJNK
46 kDa
54 kDa
JNK
46 kDa

## Slide 14
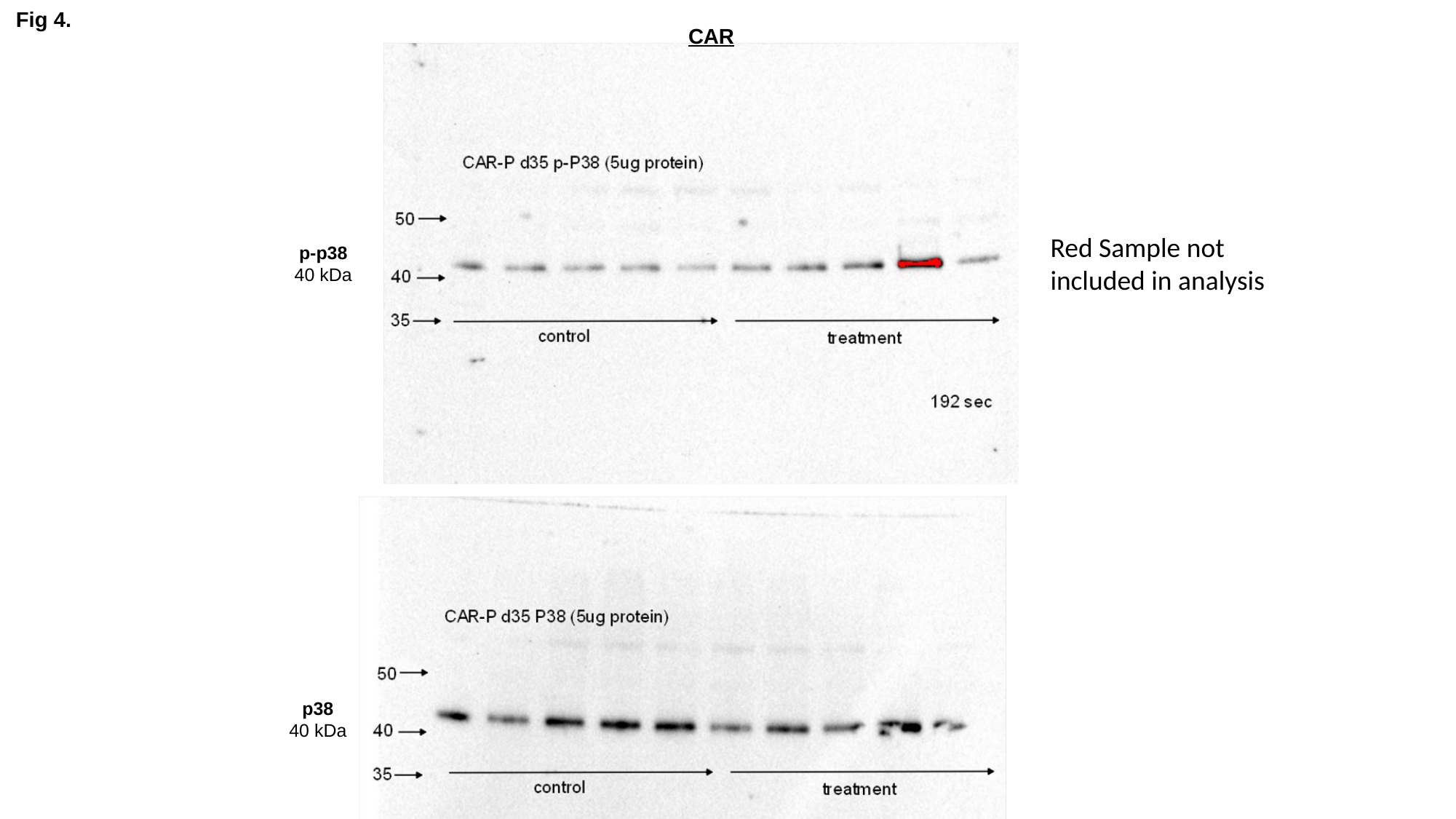

Fig 4.
CAR
Red Sample not
included in analysis
p-p38
40 kDa
p38
40 kDa
